# Evaluating heart rate coupling with Multi-dimensional Recurrence Quantification Analysis (MdRQA) and Cross-Correlation (CC): Analytical decisions and implications

**DOI:** 10.1371/journal.pone.0356801

**Published:** 2026-09-16

**Authors:** Alon Tomashin, Ilanit Gordon, Sebastian Wallot

**Affiliations:** 1 Neuroscience of Perception & Action Lab, Italian Institute of Technology, Rome, Italy; 2 Department of Psychology, Sapienza University of Rome, Rome, Italy; 3 The Gonda Multidisciplinary Brain Research Center, Bar-Ilan University, Ramat-Gan, Israel; 4 Psychology Department, Bar-Ilan University, Ramat-Gan, Israel; 5 Institute of Sustainability Psychology, Leuphana University of Lüneburg, Lüneburg, Germany; Polytechnic University of Marche: Universita Politecnica delle Marche, ITALY

## Abstract

Interpersonal heart rate coupling appears as a promising marker of group performance and coordination. However, discrepancy in methodological approaches challenges the interpretation of the results across studies. A more consistent and informed approach to preprocessing and analysis techniques could produce a more coherent picture on the value of heart rate coupling. In this manuscript, we examine the effects of different methodological decisions (resampling, analysis methods and their parameters) on the ability to capture coupling. For this purpose, we utilize both experimental dyadic heart rate data collected during a collaborative drumming task, and synthetic data with similar characteristics. Starting from preprocessing, we compare heart rate resampling window sizes, juxtapose Cross-Correlation (CC), windowed CC and Multidimensional Recurrence Quantification Analysis (MdRQA) and elaborate on the MdRQA parameter estimation and form of quantification. Our findings imply that CC as well as unembedded MdRQA recurrence rate (%REC) better distinguish coupled heart rate data from uncoupled ones, or, in other words, identify heart rate coupling. Moreover, for this objective, we suggest using Beats per Minute applying a small data-driven resampling window size, and, for MdRQA setting a moderate threshold yielding %REC between 15–25.

## 1. Introduction

The interest in using physiological and behavioral data in assessing group performance and understanding how members of a group interact and coordinate their behavior has substantially increased over the past two decades. Beyond individual traits and demographic information, researchers have been seeking a reliable biobehavioral approach to group dynamics, with an increasing focus on the temporal dimension. Accordingly, to deepen our understanding of social interactions between group members, there has been a move towards interpersonal coupling in behavioral [1,2] or (neuro-)physiological measures [3–6]. This trend entails a discussion regarding the effect of preprocessing procedures and analysis techniques on the coupling evaluation, which serves as the purpose of the current work.

One of the most prominent and pervasive features used in this context is Heart Rate (HR), which has been utilized in many studies investigating interpersonal physiological synchronization [7]. Its applicability in motion and ease of acquiring stated HR as a prominent physiological measure in joint action [8–11] – and it has been demonstrated that coordination in HR among group members is associated with group attributes such as efficacy, cohesion, and trust [7,12,13].

In the current paper, we examine the effects of pre-processing and analysis method when investigating coupling of heart rate data. We do so, because heart rate data analysis typically starts with a series of inter-event-times, the RR intervals, representing the time interval between two consecutive R wave peaks (heart beats) in an Electrocardiogram (ECG) signal. While RR intervals carry the complete variability of the heart activity, they are not equally spaced in time and hence, limits the use of such data when the aim is to correlate two or more signals, as this requires re-sampling or interpolation. On the other hand, resampled data such as Beats Per Minute (BPM) depend on the window size of the resampling interval. The main question here pertains to whether and how inter-event series such as RR intervals should be prepared to yield matching data points in order to correlate them – and calculating BPM is one way to achieve this. However, resampling and interpolation can be done in different ways, and this can potentially influence the results. For example, converting RR intervals into BPM usually entails defining a window of time over which values in the RR interval series are averaged. Then, one question is: which window sizes are principally appropriate? Such decision decisions of pre-processing can influence the results – for example removing noise from the data by heavy smoothing due to large-window averaging can remove important information from the RR signal [14].

Also, the choice of method for the calculation of physiological coupling or synchrony is important: Heart rate relies on number of internal bodily processes and is adaptable to various contexts. From a time series perspective, it results in a complex system exhibiting long-range correlations and non-stationarity [15–17]. These characteristics violate the assumption of linearity that is prominent in methods such as Cross-Correlation (CC)– a widely used method for heart rate synchrony analysis. On the other hand, there has not been a comparison to other methods, such as Multidimensional Recurrence Quantification Analysis (MdRQA), which does not make such assumptions and is also a prominent method for assessing physiological coupling. Accordingly, the difference in terms of results reached by both methods given the different options of pre-processing heart rate data has not yet been investigated.

Both methods, CC and MdRQA are frequently applied to assess behavioral and physiological synchrony, but they differ in terms of their assumptions and their outcome variables (parameter estimates). Perhaps most critically in the current context, CC assumes linearity and homogeneity of variances (e.g., [18]). Meaning that if we correlate two people’s heart rate measures, we expect that the relation between them is sufficiently captured by a straight line and that the deviations of participants’ heart rate are random and of similar magnitude, no matter whether average heart rate is low or high. However, heart rate is often non-stationary and contains long-range autocorrelations, so that the relationship between two time series differs systematically from a linear relationship [17]. Also, for heart rate, the average level of heart rate is confounded with the magnitude of its variance, so that a higher level or heart rate often goes along with higher variance as well [19]. Together, this can lead to systematic mischaracterizations of the relationship between two heart rate signals and impacts the standard errors of the estimated parameters. Also, CC is a bivariate method that can only be applied to pairs of time series (dyads). If applied to larger groups, then all possible pairwise correlations need to be run, which do not include information about higher-order group dynamics and inflate the degrees of freedom. However, CC yields a well understood parameter, the correlation coefficient ranging from −1–1, which provides information about positive and negative relationships (in- or anti-synchrony) – if the assumptions of the method are reasonably met. In case there is a violation of the linear correlation assumptions, or a substantial autocorrelation in the signal, windowed cross-correlation (WCC) is often used. By segmenting the signal into shorter windows, one can reduce the long term autocorrelation that might inflate the cross correlation values.

MdRQA is a method to correlate time series that does not assume an underlying function. Accordingly, no assumption is made about linear relationships between the data or how variance changes in a time series ([20] – see also description in the method section). Hence, non-stationary properties of heart rate time series do not violate assumptions of this method. Also, MdRQA is a multivariate method and can be used to analyze pairs of time series, but also simultaneously triples, quadruplets etc. [21]. Accordingly, correlations among the data can be directly computed on higher group-levels. However, MdRQA warrants estimation of parameters prior to analysis and has many outcome variables quantifying correlation. This leads to the problem, which of them to select for further analysis. Moreover, none of these directly distinguish between positive and negative relationships (in- and anti-synchronous correlations), as the method does not fit any function to the data that would allow for such distinctions. Accordingly, we incorporated some of these practical issues into our investigation, and compare not only CC to MdRQA, but also different variants of MdRQA with different outcome measures to help researchers make better choices when applying these methods.

Note that MdRQA and CC are not equivalent measures for coupling, they evaluate it in two different manners. CC assumes a linear function between two time series and this function can be positive or negative. In addition, it yields a metric for the strength of such a relationship. However, this is contingent on the data behaving in a linear way in the first place: CC will provide a (positive or negative) slope also if there is no such relationship in the data, even if the values of two time series cluster in a very different way. Similarly, the indicators of strength of the relationship (*r* or *R*^2^) hinge on the presence of a reasonable linear relationship between the two time series. It is entirely possible to obtain comparatively high values for *r* in the absence of a linear relationship. MdRQA on the other hand is a model-free technique, that does not fit a specific relationship to the data. Even if the relationship is complicated, not analytically definable, or changes over the course of the observation period, its outcome metrical are valid. However, this comes at the costs that certain kinds of information are currently not available using MdRQA – for example whether a relationship is positive or negative. Also, MdRQA is a multivariate technique, that can be used to analyze concordances of more than two time series at once. Still, both methods can be applied in experimental settings to investigate to what degree manipulations of context influence the degree of correlation between two time series of heart rate data. In sum, if the assumptions of CC are met (i.e., we deal with two time series that share a temporally stable linear relationship and are appropriately distributed), CC yields more detailed information. To the degree the data deviates from these assumptions, CC’s outcome metrics (the slope and *r* or *R*^2^) become intractable and MdRQA provides a better default.

Finally, as outlined above, specifically for MdRQA, there are two main questions – whether to estimate embedding parameters or not (i.e., apply time-delayed embedding; again, see Methods section for details) and which outcome variables to use in the analysis. Here we compare two popular measures and consider the effects of embedding the time series.

Our objective is evaluating the ability to discriminate coupling levels (high and low) in time series, taking into account different preprocessing of cardiac measures (various window sizes for BPM – s: S2 File), analysis method (CC, MdRQA; for WCC s. S5 File), and MdRQA analysis framework (embedding, parameter estimation, and quantification). To better understand the relationships between these variables, we not only use empirical data, but we also harness synthetic data with similar attributes to heart rate, controlling the signal-to-noise ratio and the amount of coupling within each pair of signals aiming to test the ability of each method to distinguish coupled and uncoupled pairs. In the latter part of the paper, we utilize experimental data, test linearity assumptions, and evaluate the sensitivity coupling in CC and MdRQA. For consistency, we apply the same pipeline to both synthetic and experimental datasets: testing linearity assumptions, comparing the different coupling conditions, evaluating the association between the coordination outcomes. Our goal is to provide researchers interested in heart rate coupling and synchrony with recommendations on how to best pre-process the data and choose the most useful method for their analysis.

## 2. Methods

### 2.1. Data generation

Generating heart rate-like data, we aimed to reproduce the heart rate’s fractal fluctuation, and its magnitude and to keep it in a comparable length and range to our experimental data. Kobayashi & Musha [16] were the first to discover that the heart rate of healthy adults carries a *1/f*^*α*^ (α ~ 1) fluctuation, and since then it has been reinforced in several studies. Furthermore, the fractal dimension or the α exponent was fostered as an indicator of healthy heart activity. Therefore, we first generated a vector exhibiting 1/f noise with ‘power noise’ function [22] using Matlab version 2023a (The Mathworks, Inc). Then, a linear transform was applied to reach an average RR interval of ~1000ms with a standard deviation of ~100ms. Further, we duplicated each value into a sequence with the length of its value (‘stretched time series’ for instance: [3,2,1] to [3,3,3,2,2,1]), smoothed it with a moving average of 1600ms, and rounded the results (TS1). Finally, noise was added from a Gaussian distribution with 2/3 the SD of the signal. The second RR interval of each dyad was synthesized differently depending on the condition – coupled or uncoupled– yielding a vector of different length but the same sum.

**Fig 1:**
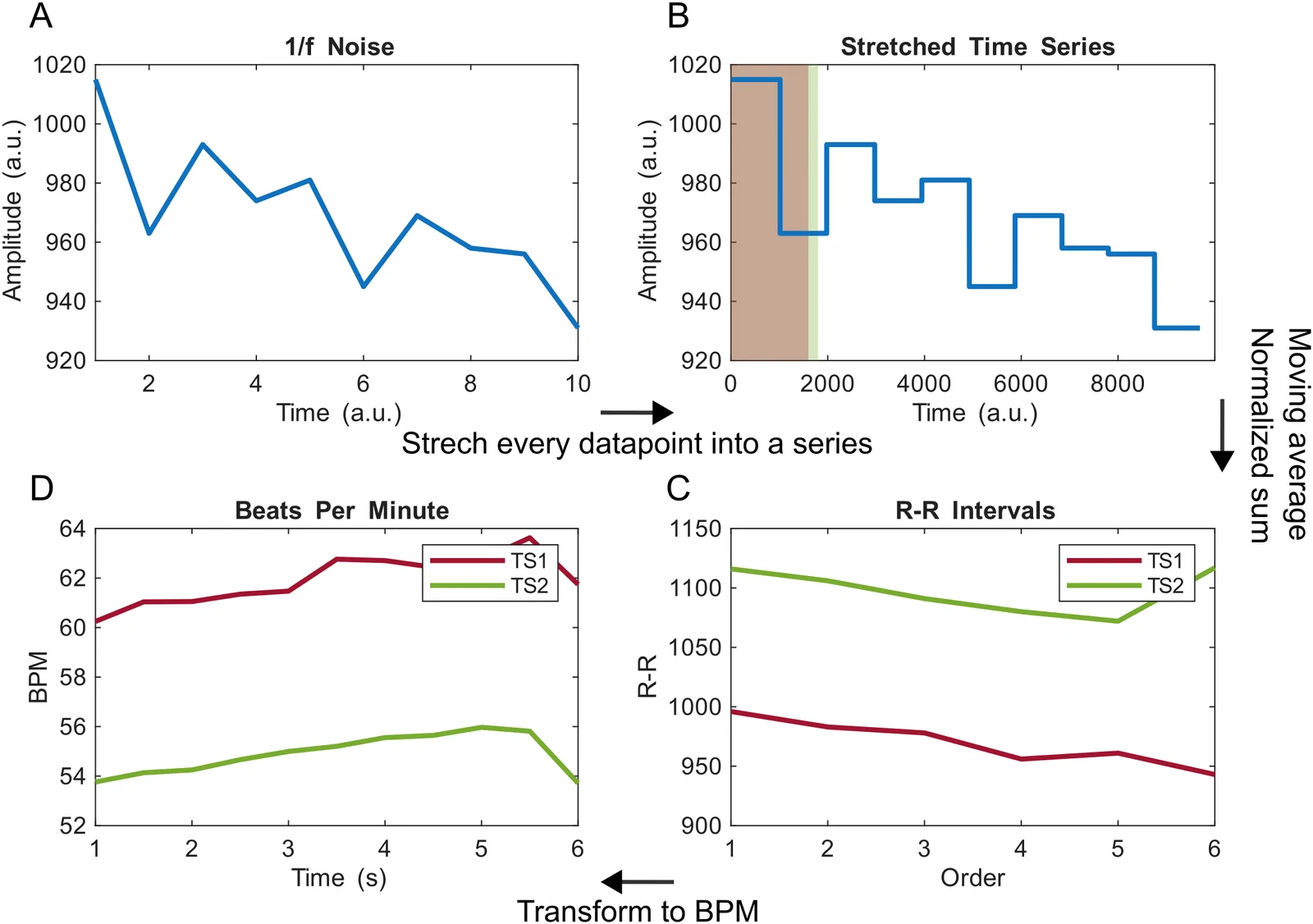
An illustration representing the 3 steps for generating the synthetic coupled RR intervals and BPM time series. SubFig A shows the time series with 1/f noise adjusted to usual RR interval scale. Then each data point is duplicated into a vector the length of its value (B); as a reference, the length of B equals to the sum of A. A moving average is applied on the stretched time series which is then rounded resulting in RR intervals (C), while under the ‘coupled’ conditions a different window size is used for ts2 to generate coupled yet not equal time series pair. To compute BPM, we applied non overlapping windows of 512ms.

**Fig 2:**
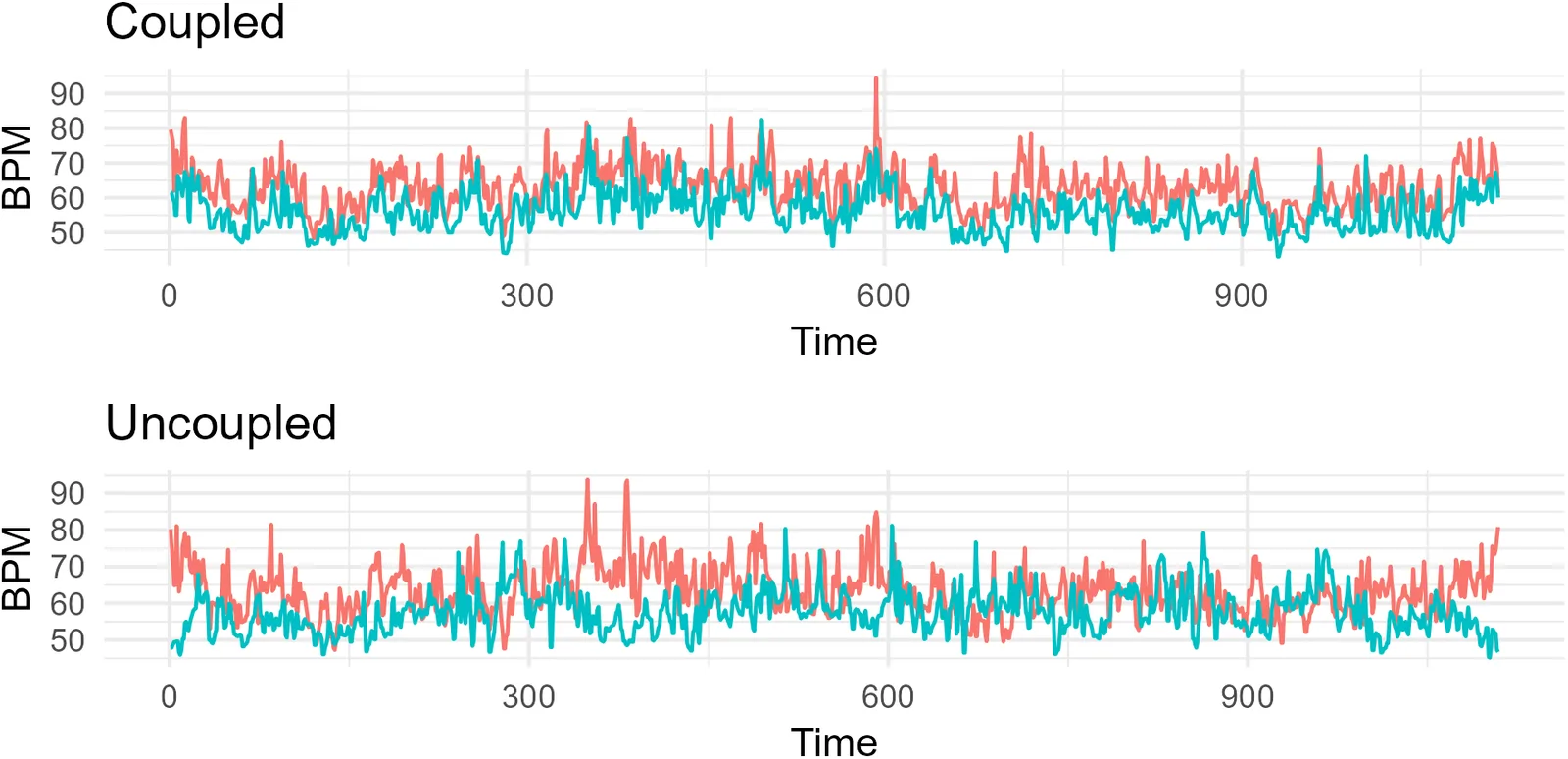
Exemplary BPM time series of the 2 conditions – two BPM-series that are coupled, and two series that are uncoupled.

The first variable provides two levels of coupling within the pair of time series with 100 iterations for each level. In the ‘uncoupled’ condition, the second time series (TS2) is generated randomly in the same manner as the first. To introduce coupling, under the ‘coupled’ condition, the second time series was drawn from the same 1/f noise vector (Fig 1A) while we applied a moving average of 1800ms instead of 1600ms on the first stretched time series, added a random Gaussian noise with the same 2:3 signal to noise ratio (SNR), and then normalized it to the sum of TS1 to yield the same time span. Additionally, we converted the RR intervals into BPM with non-overlapping windows of 512ms resulting in BPM in 0.512 seconds resolution [14]. This value, equals to the shortest RR interval in the series was chosen based on a comparison described in details in S2 File. There, we contrasted different data-driven resampling window sizes, ranging up to two times the maximum RR interval in the series. Within this range, the different window sizes did not systematically moderate the results. For that reason, the shortest RR interval was preferable since it retains all the variability in the data (see [14]). Fig 2 shows example time series for the coupled and uncoupled conditions.

The rationale for setting these parameters for the synthetic data was to produce data that are in the average range of accepted definitions of human heart rate and empirical observations. For our synthetic data, these are: mean BPM = 81.3, SD BPM = 5.4, Hurst exponent BPM = 0.95 – these are in the average range of healthy human heart rate, see [17,23]. For the experimental data presented in this paper, the respective descriptives are mean BPM = 58.4, SD BPM = 5.9, Hurst exponent BPM = 0.91. Regarding the addition of random noise to our synthetic data, we chose a SNR that would yield a similar overall range (SD) compared to the experimental data, and at the same time not de-correlate the time series to yield a hurst exponents close to 1. Note, however, that all data were z-transformed anyway before being subjected to the respective analyses.

Furthermore, to introduce temporal dynamics in the coupling strength, as expected in heart rate data, we generated time series that are intermittently coupled (for further information, s. S4 File).

### 2.2. Experimental data

To explore the association between the cardiac measurements, as well as the different coordination outcomes, we selected a phenomenon that have already been associated with cardiac synchrony – drumming in groups [24,25]. Aiming for a comparable sample size to the aforementioned study [23], our sample consisted of 50 pairs of participants (72% females; average age = 23.1, *SD* = 2.8). Participants’ heart rate was monitored during a collaborative drumming in-lab experiment; 2 groups were discarded due to incomplete or missing data. In this task, participants were asked to drum together, each one on their assigned pad, and improvise in search of musical patterns and were demonstrated a famous beat as an inspiration – “We Will Rock You” (May, 1977).

Electrocardiography was measured at 500 Hz simultaneously from both participants by MindWare Cardiograph Impedance Mobile devices (MindWare technologies, Gahanna, Ohio). The signal was then amplified, filtered and preprocessed by trained research assistants using MindWare Technology’s HRV application (version 3.1.5) according to the manufacturer guidelines resulting in RR intervals. This signal was transformed into BPM in the same manner explained above with non-overlapping windows of 410ms (i.e., the minimum RR interval in the dataset).

In this manuscript, we do not intend to investigate the interpersonal physiological coordination through music making. Rather, we utilize this dataset to better understand the outcomes of MdRQA and CC, further examine the synthetic data results, and ideally, to provide guidelines regarding the decisions taken throughout heart rate coordination analysis.

### 2.3. Ethics

The drumming experiment was approved by the ethics board of Bar-Ilan University (approval number: 2021/41EX). There was no deceit in the study and written informed consent was obtained from all participants. Participants were recruited between November 2021 and October 2025.

### 2.4. Analysis techniques

As stated earlier, we compared cross-correlation at lag0 or Pearson’s correlation with Multi-dimensional Recurrence Quantification Analysis (MdRQA). Some researchers apply variations of these analysis techniques such as WCC (S5 File), Cross or Joint Recurrence Quantification Analysis, et cetera. We aimed to further understand the outcomes and the interpretations of the methods in analyzing HR and therefore focused on the basic versions.

### 2.5. MdRQA

Multi-dimensional Recurrence Quantification Analysis (MdRQA, [21]) is a recurrence-based method to analyze non-linear time series derived from the uni-dimensional RQA [26]. It utilizes the Recurrence Plot (RP) to capture various aspects of the time series dynamics, basically similarity of the dynamics over time. The first step in RQA is phase-space reconstruction, based on Takens’ theorem [27], which suggests that the dynamics of the whole system can be recovered from a single observable by the method of delayed embeddings. MdRQA is a multivariate extension of RQA and utilizes 2 or more measured variables to reconstruct the phase-space and hence enables investigation of the coordination within groups (i.e., multiple time series) in time.

In essence, to conduct MdRQA one first estimates the time-delayed embedding parameters, delay and embedding dimension, by applying Multidimensional Average Mutual Information and False-Nearest Neighbors (MdFNN and MdDelay; [28]). Having constructed the phase-space, a distance matrix is formed between each pair of data points. For quantification purposes, this matrix is then dichotomized by applying a threshold (*r*) forming a Recurrence Plot (RP) in which a distance lower than *r* defines a pair of points as recurrent. Usually, the value of *r* is assigned to yield a low, yet sufficient percentage of recurrence points to evaluate the recurrence dynamics. For a detailed tutorial on parameter estimation in RQA and MdRQA, see Wallot & Leonardi [29].

**Fig 3:**
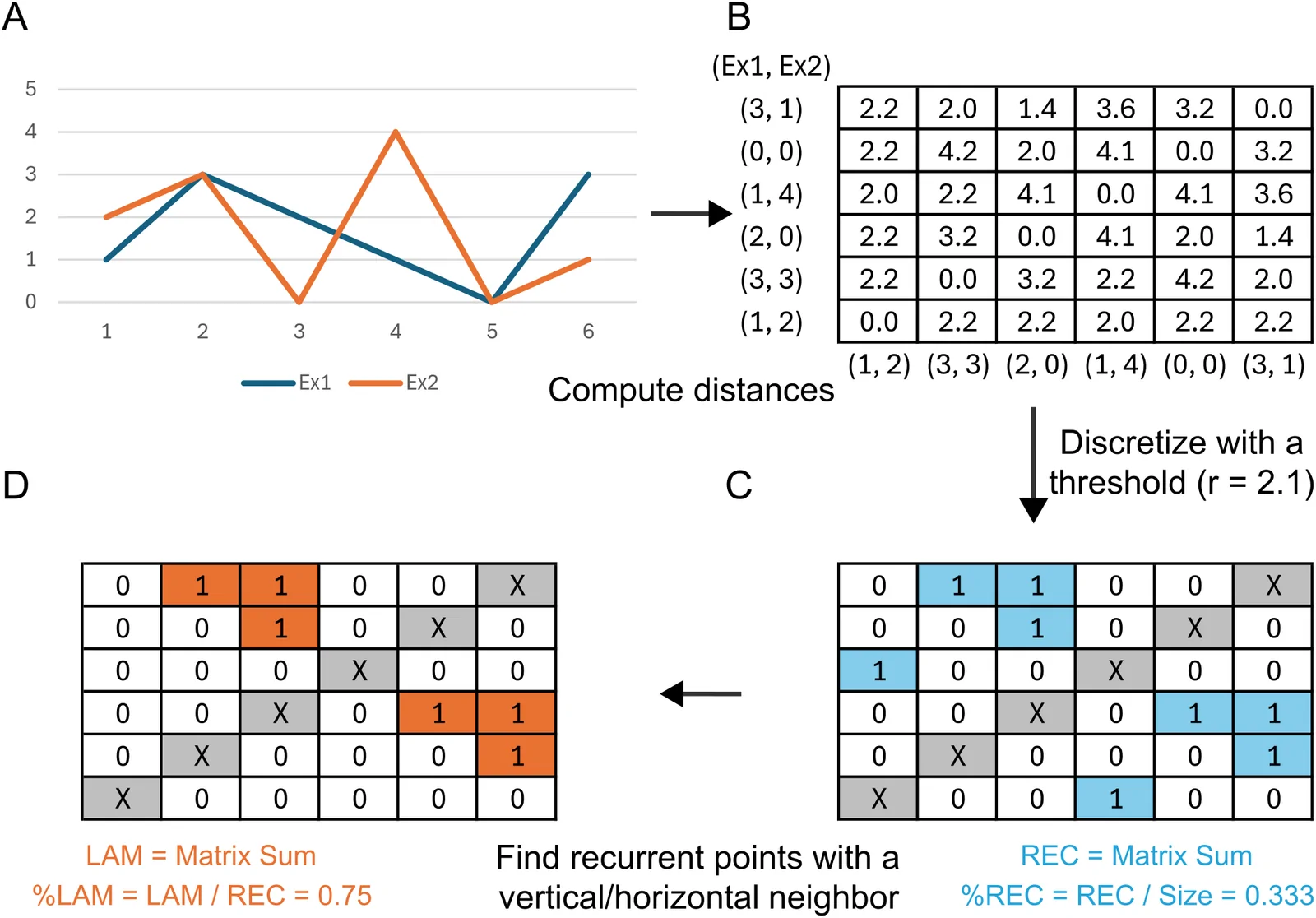
An illustration representing the extraction of %REC and %LAM through unembedded MdRQA. SubFig A shows two time series. Based on them, the distance matrix is computed with Euclidean distance between each pair of data points (B). The distance matrix is then discretized according to a certain threshold (r) yielding the recurrence plot (C) in which each blue square represents a recurrent point – for this toy example the threshold was chosen so it provides a high enough number of recurrent points. Among the recurrent points, only the ones with a vertical or horizontal neighboring recurrent points were painted in orange (D) to represent the Laminarity.

Several measures could be drawn from the RP such as the percentage of recurrent points (%REC) or the laminarity (%LAM) – percentage of recurrent points that have vertical or horizontal neighbors (Fig 3). Additionally, other measures describe the lengths of recurrent lines in the matrix. Focusing on physiological data exhibiting 1/f characteristics [16] with long-term autocorrelation we anticipated non uniform structures with sporadic patches of recurrence. Hence, we chose %LAM to capture these patterns alongside %REC as a reference. Briefly, %REC captures element-wise similarity between two time series, and counts the number of individual data points that are either similar (i.e., recurrence) or dissimilar (i.e., non-recurrent) across two or more time series and provides a percentage of recurrent points compared to total number of points possible. %LAM on the other hand captures whole clusters of data points between two or more time series that group a pattern. As heart-rate data is not random but exhibits long-range correlations over time [17], some data points tend to group together in highly correlated clusters. Those from clusters of recurrences between time series, which are captured by %LAM. So, while high %REC tells us that there is a strong correlation among the time series in terms of the individual data points, high %LAM tells such that much of that correlation is not only due to individual data points that are similar, but rather that correlation is due to the formation of correlated clusters between the time series formed by multiple adjacent data points. For further information regarding the method refer elsewhere [21].

MdRQA was performed on z-scored bivariate BPM time series in two manners – with and without phase-space reconstruction by means of time-delayed embedding. No phase-space reconstruction means that the recurrence measures were calculated on the raw data. Using embedding of the data, it is possible to extract additional information from the measures time series by including information about autocorrelation patterns in the data. In model systems, this leads to a more accurate depiction of the dynamics or the coupling among time series [20] but does not necessarily lead to superior results for empirical data, particularly if that data is very noisy. We used average mutual information and false-nearest neighbor functions to estimate delay and embedding dimension parameters, using the first local minimum of each function. The process of parameter estimation is explained in detail in Wallot & Leonardi [29], which we followed here. For the embedded version, we estimated the delay as 2 and the embedding dimension parameters as 25 (synthetic) and 15 (experimental) relying on MdDelay and MdFNN ([28].

We then determined thresholds yielding ~15% recurrence for each analysis (Synthetic: *r*_*Unembedded*_ = 0.7, *r*_*Embedded*_ = 8, Experimental: *r*_*Unembedded*_ = 0.7, *r*_*Embedded*_ = 5). In S3 File, we compare various threshold values (or radii), the respective MdRQA outcomes and the resulting differentiation between the coupling conditions.

## 3. Results

### 3.1. Synthetic data

First, we tested which percentage of our dataset meets the linear correlation assumptions – homoscedasticity (Breusch-Pagan test), linearity, and normality of residuals (Shapiro-Wilk test). Table 1 indicates that a substantial portion of the data sets violate some of the assumptions of linear correlation. Particularly, BPM data violates the normality of the residuals’ assumption in most cases.

**Table 1:** Percentage of the time series which significantly violate the assumptions linear correlation. The alpha-level was set to 0.05 – accordingly, by chance we expect 5% of the data sets to fail the tests.

Test/Measure	BPM
Homoscedasticity	64%
Linearity	26%
Normality of residuals	99.5%

Then, we compared the coupling conditions within each analysis unit using Welch’s independent samples *t*-test. As shown in Table 2, overall, all measures distinguish ‘coupled’ from ‘uncoupled’ – also when taking into account Bonferroni correction for multiple tests (α = 0.01).

**Table 2:** Welch’s t-test comparing ‘coupled’ and ‘uncoupled’ conditions. U: unembedded, E: embedded.

Coupled-Uncoupled	Statistic	df	p	Cohen’s d	Mean (Coupled)	Mean (Uncoupled)
CC BPM	62.044	151	< .001	8.77	0.62	0.006
%REC(U) BPM	35.738	151	< .001	5.05	15.5	12.4
%LAM(U) BPM	8.067	185	< .001	1.14	81.7	79.5
%REC(E) BPM	17.814	179	< .001	2.52	23.5	15.2
%LAM(E) BPM	11.652	192	< .001	1.65	93.8	90.9

Note. H_a_ μ _Coupled_ ≠ μ _Uncoupled._

Further, we were interested in the correlation between the measures within each coupling condition. The first column of Table 3 shows a strong correlation between CC and unembedded %REC under the ‘coupled’ condition that does not pertain for the ‘uncoupled’ condition (Table 4). One can also notice a strong link between all the MdRQA measures regardless of the coupling level.

**Table 3:** A correlation matrix summarizing the associations between the different coordination measures under the ‘coupled’ condition. U: unembedded, E: embedded.

Coupled	CC BPM	%REC(U) BPM	%LAM(U) BPM	%REC(E) BPM	%LAM(E) BPM
CC BPM	—				
%REC(U) BPM	0.662***	—			
%LAM(U) BPM	0.223*	0.583***	—		
%REC(E) BPM	0.101	0.637***	0.6***	—	
%LAM(E) BPM	0.179	0.472***	0.6***	0.631***	—

Note. * p < .05, ** p < .01, *** p < .001

**Table 4:** A correlation matrix summarizing the associations between the different coordination measures under the ‘uncoupled’ condition. U: unembedded, E: embedded.

Uncoupled	CC BPM	%REC(U) BPM	%LAM(U) BPM	%REC(E) BPM	%LAM(E) BPM
CC BPM	—				
%REC(U) BPM	0.061	—			
%LAM(U) BPM	0.072	0.359***	—		
%REC(E) BPM	0.225*	0.537***	0.395***	—	
%LAM(E) BPM	0.157	0.306**	0.541***	0.652***	—

*Note.* * p < .05, ** p < .01, *** p < .001.

Additional analyses that investigated the effects of noise and embedding in MdRQA show that, unsurprisingly, noise generally reduced the effect size of comparing coupled vs. uncoupled data for CC and well as for MdRQA. However, the recurrence measure of %LAM was particularly sensitive to noise in general, and embedding also generally reduced the sensitivity of both recurrence measures to detect differences in coupling (a deeper investigation of the limitation of %LAM is provided in S1 File). Furthermore, we compared competence of WCC and CC to distinguish coupled and uncoupled signals (S5 File). The results were similar and no method was more favorable than the other [14,29].

In summary, our findings indicate that first, there is evidence that heart rate-like data is not appropriate for cross-correlation, at least when analyzed at full length (without windows). Second, in distinguishing pairs of time series based on coupling levels, BPM is a more reliable across coordination measurements. When analyzing BPM, all MdRQA measures as well as CC yield the same pattern of results: ‘coupled’ > ‘uncoupled’. However, only %REC from unembedded MdRQA was correlated with CC suggesting some overlap between the measures when the two time series are coupled. Further, although MdRQA measures, both embedded and unembedded, are positively correlated, the moderate degree of their correlation suggests that they are not interchangeable.

### 3.2. Experimental data

In accordance with the synthetic analysis, we present linear assumption tests for experimental BPM. With an α = 5%, the data break the homoscedasticity and normality of residuals assumptions underlying linear correlation to high extent (70.8% and 93.8% correspondingly) while the linearity assumption is reserved (12.5% violation among the pairs).

As an equivalent to the conditions in the simulated data, here we investigated participants who interacted together, in comparison to false-pair surrogates. For every dyad, we replaced the second participant with one randomly selected from a different group to create a false pair considering the participants’ positions in the lab (i.e., participant who sat on the right side of the drum set was matched with one that sat on the left side). Then, we evaluated the heart rate (BPM) coordination of the ‘Real’ and the ‘Surrogate’ dyads using MdRQA and CC and compared the two conditions with a paired sample *t*-test. A significantly (Bonferroni corrected α = 0.01) higher coordination was found among the Real dyads only using CC (*t*(47) = 4.93, *p* < 0.001, Cohen’s *d* = 0.71) and unembedded %REC (*t*(47) = 2.86, *p* = 0.006, Cohen’s *d* = 0.41) while *t*he effect in %REC (embedded) was nearly significant (*t*(47) = 2.34, *p* = 0.024, Cohen’s *d* = 0.34) – see fur*t*her descriptives in Table 5. Both embedded and unembedded %LAM were insignificant in distinguishing between the conditions (*t* < 0.46, *p* > 0.65).

**Table 5:** Mean and standard deviations of the correlation coefficient, unembedded and embedded %REC for the two conditions.

	Condition	CC BPM	%REC(U) BPM	%REC(E) BPM
Mean	Real	0.178	14.88	17.23
	Sur	0.008	13.8	14.81
Standard deviation	Real	0.154	1.96	9.34
	Sur	0.24	3.42	5.88

Among the Real pairs (Table 6), we found significant correlations between CC and MdRQA measures whilst these correlations vanished within the Surrogate pairs (Table 7). Similar to the synthetic analysis, MdRQA measures are positively correlated, here, except the association between unembedded %REC and embedded %LAM.

**Table 6:** A correlation matrix summarizing the associations between the different coordination measures considering Real pairs. U: unembedded, E: embedded.

Real	CC BPM	%REC(U) BPM	%LAM(U) BPM	%REC(E) BPM	%LAM(E) BPM
CC BPM	—				
%REC(U) BPM	0.557***	—			
%LAM(U) BPM	0.515***	0.504***	—		
%REC(E) BPM	0.549***	0.83***	0.685***	—	
%LAM(E) BPM	0.312*	0.279	0.66***	0.529***	—

Note. * p < .05, ** p < .01, *** p < .001.

**Table 7:** A correlation matrix summarizing the associations between the different coordination measures considering Surrogate pairs. U: unembedded, E: embedded.

Surrogate	CC BPM	%REC(U) BPM	%LAM(U) BPM	%REC(E) BPM	%LAM(E) BPM
CC BPM	—				
%REC(U) BPM	-0.285	—			
%LAM(U) BPM	-0.132	0.452**	—		
%REC(E) BPM	-0.284	0.726***	0.611***	—	
%LAM(E) BPM	0.015	0.084	0.512***	0.496***	—

*Note.* * p < .05, ** p < .01, *** p < .001.

Just as with the simulated data, additional analyses investigating variations of the resampling window size of the RR intervals to BPM conversion – again using as the smallest windows size a window equal to the shortest RR interval in the series and using as the largest window size a window equal two times the maximum RR interval in the series – did not affect the results (s. S2 File). Also, and similarly to the synthetic data, higher radii (15–25% recurrence) increased the sensitivity of MdRQA to distinguish between the original (i.e., coupled) and surrogate (i.e., uncoupled) data (s. S3 File).

Overall, the experimental data analysis replicates the findings of the previous analysis. It supports the positive association between CC and MdRQA measures among coupled time series; in this case without limiting it to unembedded %REC. Nevertheless, it calls into question the suitability of %LAM in discriminating coupled BPM time series from uncoupled or weakly coupled ones.

## 4. Conclusions

In this paper, we examined the consequences of preprocessing and analysis decisions on interpersonal heart rate data analysis. Starting from preprocessing, the resampling window when converting RR intervals to BPM, does not substantially affect the results (at least not within the range of minimum RR interval to maximum RR interval multiplied by 2; s. S1 File). Hence, we recommend applying a data-driven approach and choosing the shortest RR interval within the sample as the size of the resampling window (see also [14]. While this bottom-up approach may complicate the aggregation of multiple independent studies – since no common window size is used – it avoids imposing benchmarks that may be inappropriate for different populations or contexts with differing heart rate. As long as the same preprocessing decisions are applied, the results will be generalizable.

Note that this does not imply that BPM is generally superior to other measures of heart rate. Raw RR intervals can also be resampled and interpolated to produce matching time series. Here, we used BPM as one means of achieving evenly spaced time series. In any case, our results suggest that resampling or interpolation can be done based on the properties of the time series at hand, using rather small window sizes so as not average-out too much of the fluctuations in the data.

Considering analysis decisions in MdRQA, we found that not embedding the data is favorable when focusing on the coupling levels. Moreover, examining the variation of the radius parameter suggests that medium and large radii are preferable yielding somewhat higher recurrence rates are preferable (here in the range of ~15, and ~25%REC; s. S1 File).

Finally, when comparing methods (CC and MdRQA) we found that both CC and %REC perform well at discriminating coupling levels. This goes for the artificial cases of continuous coupling, and discrete changes in coupling, as well as for experimental time series. However, as mentioned earlier, our simulations might have favored types of coupling that are more amendable to of CC and there are surely stronger violations of the assumption of linearity as the case of discrete coupling changes. In general, if the assumptions of CC are violated, the standard errors might be inflated, but specifically the slopes might not be misleading. They might suggest types of relationships that are not in the data and thus lead to faulty conclusions.

In line with previous research considering %LAM as a measure for fluctuation type [30], our examinations suggest that %LAM in general, but also %REC from *embedded* MdRQA are more sensitive to noise levels or fractal dimensionality of the data. For this reason, probably, they do not distinguish the coupling conditions with the same efficiency and %REC from *un*embedded MdRQA should be preferred as outcome measure (s. S3 File).

Our findings raise another interesting point regarding the association between CC and %REC from unembedded MdRQA: Both synthetic and experimental data indicate that the correlation between the two coordination measurements appears only when the two time series are coupled. That is, %REC from unembedded MdRQA is not a mere equivalent to CC, instead, the association between the outcomes of the two methods stems from the coupling levels in the data. We think that the origin of this disparity might stem from the violation of linearity that is more prevalent with uncoupled time series. In these uncoupled cases, CC outcomes are more random and cannot be meaningfully interpreted. Since linearity is often violated in heart rate data and, researchers usually deal with low correlations between different heart-rate signals.

In general, violations of the assumptions of linearity, homoscedasticity and normality can undermine the validity of cross-correlation analysis. If linearity is violated, CCF might captures only a distorted or incomplete picture of the relationship (i.e., just providing the best linear fit to data whose relationship follows a different pattern), potentially missing important (nonlinear) dependencies. Heteroscedasticity (non-constant variance) further compromises interpretation by inflating or attenuating correlations at specific lags, leading to spurious peaks and unreliable confidence intervals. Deviations from normality of residuals do usually not bias the parameter estimates in large enough samples but can artificially inflate or deflate the estimate of standard errors and thus invalidate standard inferential procedures, increasing the risk of incorrect significance assessments, particularly in smaller samples or in the presence of outliers. Together, these violations can result in misleading conclusions about the strength and statistical significance of relationships between time series.

Hence, in cases of unknown coupling conditions and strong violation of the linearity assumption, we would hence recommend utilizing unembedded %REC instead of CC – or use both analysis procedures together to look for convergent effects. If the heart rate data exhibits strong long-range correlations – which is often the case [17] and might be one of the primary causes of the violation of assumptions in CC – applying fractional differencing [31] before running CC might be one way to improve the situation regarding violations of linearity and violation of homogeneity of variances.

Furthermore, although our synthetic and experimental analyses yielded similar results, the synthetic sample incorporated continuous coupling with same strength which might not represent natural association between two heart rate signals, where coupling levels might vary over time [32]. Nevertheless, in order to address this issue, we conducted a separate analysis which includes intermittent coupling that could better represent the synchronization dynamics between two heart rate time series (S4 File). Results from this analysis were in line with our findings where CC and unembedded %REC differentiate to the largest extent between stably coupled, intermittently coupled and uncoupled time series. As already mentioned, coupled time series can be generated in various ways, producing different forms of coupling that may be preferentially detected by specific analytical approaches. The manner we applied (described in detail in Methods) might be better amendable to correlation-based analyses than to other types of coupling. However, since we utilize experimental data as well, which showed similar trends, our claim that coupling in BPM data is better represented by CC and %REC (unembedded) is well supported.

To sum up, based on both synthetic and experimental data analysis, our recommendations to quantifying coupling in heart rate: 1. Use BPM (or similarly resampled data), preferably with a low data-driven resampling window size). 2. Use CC (particularly in cases where linearity is seldom or not strongly violated) or WCC, and %REC from *unembedded* MdRQA as outcome measures of association between heart rate signals. 3. Specifically, for MdRQA: When investigating heart rate coupling, do not apply embedding (if you have no specific technical or theoretical reason to do so) and use a radius parameter that yields ~15% recurrence, on average.

## Supporting information

S1 File Effects of noise level and embedding on coordination measures of MdRQA. (DOCX)

S2 File Different BPM resampling window sizes. (DOCX)

S3 File Different radii in MdRQA. (DOCX)

S4 File Intermittent Coupling. (DOCX)

S5 File Windowed Cross Correlation (WCC). (DOCX)
