# Supplementary material for: Evaluating heart rate coupling with Multi-dimensional Recurrence Quantification Analysis (MdRQA) and Cross-Correlation (CC): Analytical decisions and implications
Source: PLoS One. 2026 Sep 16;21(9):e0356801. doi: 10.1371/journal.pone.0356801 (PMC13580983; doi:10.1371/journal.pone.0356801)
Supplement: Supplementary file 1 [file pone.0356801.s001.docx]

S1. Effects of noise level and embedding on coordination measures of MdRQA

As mentioned before, our results suggest that %LAM is an incompatible measure for coupling at least as presented between BPM time series. Hence, we were interested in what characteristics of the data %LAM is sensitive to. Based on previous literature connecting %LAM and fractal dimension (Tomashin, Leonardi, et al., 2022), we expected that this measure focuses on the dynamics of the fluctuation within the time series. To examine this hypothesis, we added another factor to our synthetic data analysis – noise. In our original analysis, Gaussian noise (SNR 3:2) was added to the time series, while the new conditions include no additional noise, resulting in 2 by 2 design: coupled/uncoupled, with/without noise. Importantly, this noise component is artificial and should not be confused with power line noise in real ECG recording.

Uncorrelated noise decreases the correlation between the time series only if the time series are coupled. Furthermore, it flattens the 1/f^α^ fluctuation, or, in other words, creates more random time series. Therefore, if a measure attends to the coupling or correlation level, it will distinguish between the two noise conditions among the coupled but not among the uncoupled pairs. Alternatively, a measure that focuses on the temporal dynamics of the time series will differentiate noise level under both coupled and uncoupled conditions. To examine these hypotheses, hen, we compared the coupling conditions within each analysis measure using Gamez-Howell post-hoc tests and Bonferroni correction for multiple comparisons within each measure (6 comparisons). As shown in Figure 4, CC and unembedded %REC yields higher correlation coefficient for ‘coupled’ over ‘Coupled + Noise’ while no significant difference appears when introducing noise to the ‘Uncoupled’ condition.

Figure S1.1. Violin plots demonstrating a comparison of the Pearson's coefficients (A) and unembedded %REC (B) computed on TS1&TS2 between the different coupling conditions. On panels A and B, the mean of each condition is presented while the significance of the post-hoc comparisons corrected for 6 comparisons is indicated by the horizontal bars. The coordination measures show a similar pattern of results: ‘coupled’ > ‘coupled + noise’ > ‘uncoupled’ = ‘uncoupled + noise’.

Inspecting embedded %REC and %LAM (embedded and unembedded) one observes significant differences between the noise condition also when the time series are uncoupled (Figure 5). It suggests that these measures are sensitive to noise as a fluctuation component. Furthermore, all measures demonstrated below differentiate coupling levels within each noise condition, meaning they also capture coupling between the time series. Another interesting point arising from the comparison between ‘uncoupled’ and ‘coupled + noise’ in %LAM and embedded %REC. While %LAM is higher for ‘uncoupled’, the two conditions do not differ in embedded %REC. It could imply that %LAM gives more weight to the fluctuation dynamics, although embedded %REC gives similar weights to the two components – dynamics and coupling.

Figure S1.2. Violin plots demonstrating a comparison of the unembedded %LAM (A), embedded %REC (B) embedded %LAM (B) computed on TS1&TS2 between the different coupling conditions. On panels A, B and C, the mean of each condition is presented while the significance of the post-hoc comparisons corrected for 6 comparisons are indicated by the horizontal bars. The %LAM measures show a similar pattern of results: ‘coupled’ > ‘uncoupled’ > ‘coupled + noise’ > ‘uncoupled + noise’. However, in embedded %REC ‘uncoupled’ and ‘coupled + noise’ did not differ.
