# Supplementary material for: Evaluating heart rate coupling with Multi-dimensional Recurrence Quantification Analysis (MdRQA) and Cross-Correlation (CC): Analytical decisions and implications
Source: PLoS One. 2026 Sep 16;21(9):e0356801. doi: 10.1371/journal.pone.0356801 (PMC13580983; doi:10.1371/journal.pone.0356801)
Supplement: Supplementary file 2 [file pone.0356801.s002.docx]

S2. Different BPM resampling window sizes

Another point of discussion when addressing cardiac measures preprocessing is the window size used to convert the RR intervals into BPM. For this purpose, we conducted the same analyses described in the paper on BPM generated with 3 window sizes (synthetic: 512, 1031, 1858ms; experimental: 410, 754, 1468ms). Our approach to choosing window sizes is data driven, the lowest one (‘min’) is determined by the minimum RR interval value, another is the average interval (‘avg’), and the highest is the maximum RR interval multiplied by 2 (‘max2’). It is worth noting that besides the effect window size has on the fluctuation, a larger window yields a shorter time series carrying less information.

To test the differences in the coordination levels between ‘coupled’ and ‘uncoupled’ synthetic heart-rate-like data we conducted a linear mixed model for each coordination measure. We were interested in the interaction between condition and window size (WS), especially whether WS moderates the ability to distinguish between the two coupling conditions. All models yielded a significant interaction (*p* < 0.001). However, when observing the moderation effect of WS all the simple effects of the coupling condition remain significant between the WS levels. Meaning, WS changes the effect size but not its direction and as can indicated in Figure 6, there is no one WS that is preferable across all the measures. While max2 WS distinguishes better coupling levels better within unembedded MdRQA measures and CC, avg and min are preferable when applying embedded MdRQA.

Figure S2.1. Distinguishing coupling conditions with different WS (512, 1031, 1858ms) within the synthetic data. The differences between the conditions are larger when applying a large window size for CC and unembed %REC and %LAM. Notwithstanding, min WS shows a larger difference between the conditions for embedded %REC and avg WS yields the largest difference in embedded %LAM. min: minimum RR, avg: average RR, max2: maximum RR multiplied by 2.

All in all, in both analyses the competence of the measurement to distinguish coupling levels did not change due to window size (significant results remained significant for all window sizes). For that reason, we chose the window size which affects the original RR signal the least, that is the lowest window size.
