# Supplementary material for: Evaluating heart rate coupling with Multi-dimensional Recurrence Quantification Analysis (MdRQA) and Cross-Correlation (CC): Analytical decisions and implications
Source: PLoS One. 2026 Sep 16;21(9):e0356801. doi: 10.1371/journal.pone.0356801 (PMC13580983; doi:10.1371/journal.pone.0356801)
Supplement: Supplementary file 3 [file pone.0356801.s003.docx]

S3. Different radii in MdRQA

One more consideration when conducting MdRQA is the radius estimation. As there are no current techniques to estimate a suitable radius for the data, researchers usually choose a radius, based on a “rule of thumb”, yielding a %REC of 1-5%. In the current analysis, we investigated the effect of the radius on our results, specifically on the discrimination between coupled and uncoupled time series. For this purpose, we used 3 radii per analysis (small, medium and large) yielding a %REC of ~5, ~15 and ~25 accordingly (Table 8).

Table S3.1. Different radii and their relative %REC and %LAM with and without embedding for both synthetic (syn.) and experimental (exp.).

| **r** | **r(U)** | | **%REC(U)** | | **%LAM(U)** | | **r(E)** | | **%REC(E)** | | **%LAM(E)** | |
| --- | --- | --- | --- | --- | --- | --- | --- | --- | --- | --- | --- | --- |
|  | Syn. | Exp. | Syn. | Exp. | Syn. | Exp. | Syn. | Exp. | Syn. | Exp. | Syn. | Exp. |
| Small | 0.4 | 0.4 | 4.89 | 5.17 | 57.5 | 49.6 | 7 | 4 | 6.32 | 3.93 | 86.5 | 58.5 |
| Medium | 0.7 | 0.7 | 13.9 | 14.4 | 80.6 | 73.6 | 8 | 5 | 19.3 | 11.7 | 92.3 | 75.4 |
| Large | 0.9 | 0.9 | 21.7 | 22 | 88.2 | 82.2 | 8.5 | 6 | 28.7 | 26.3 | 94.2 | 87 |
|  | | | | | | | | | | | | |

Similarly to the window size comparison, to study the effects of r on the MdRQA measures’ competence to distinguish the different coupling levels of the synthetic data we conducted 4 mixed models – one for each measure. Our main objective was the interaction between the radius and the coupling level. All the models yielded a significant interaction (*p* < 0.001) and hence we further inspected the simple effects of coupling for each radius. Figure 7 indicates that larger radii distinguish the conditions better when measuring %REC, while the opposite pattern appears for %LAM. For this reason, our decision was narrowed to the medium and the large radii and choosing the medium radius was not of particular benefit over the large radius.

Figure S3.1. Distinguishing coupling conditions with different radii. The differences between the conditions are larger when applying larger radii for %REC (embedded and unembedded). In contrast, a small radius yielded a larger effect size in %LAM.
