# Supplementary material for: Evaluating heart rate coupling with Multi-dimensional Recurrence Quantification Analysis (MdRQA) and Cross-Correlation (CC): Analytical decisions and implications
Source: PLoS One. 2026 Sep 16;21(9):e0356801. doi: 10.1371/journal.pone.0356801 (PMC13580983; doi:10.1371/journal.pone.0356801)
Supplement: Supplementary file 4 [file pone.0356801.s004.docx]

S4. Intermittent Coupling

While our experimental data provides us with the most real-life example for interpersonal coupling with its complexity and flexibility over time, we sought to create a condition within our synthetic dataset that better captures the intricacies of a realistic phenomenon of interpersonal synchrony. Hence, beyond coupled and uncoupled conditions we add intermittent coupling (IC). That is, two time series that are coupled 50% of the time and uncoupled during the rest. Keeping in mind that this tendency to go in and out of synchrony could occur more than once throughout an experiment, we alternated between coupling and uncoupling 10 times over the length of the time series. Practically, we generated TS2 in 10 segments of 10% of the total length deriving from TS2 of the coupled and the uncoupled condition in the following manner: the first segment was taken from the coupled condition, the second was taken from the uncoupled condition and so forth. Consequently, in IC, TS2 was coupled with TS1 half the time with 9 points of alternation between the coupling levels.

To examine the ability of the various analysis techniques to differentiate the three conditions: stably coupled (SC), intermittently coupled (IC) and uncoupled (UC), we performed a One-Way ANOVA (Welch’s) and Games-Howell post-hoc tests between all the pairs. In accordance with the findings presented in Table 9, the pair-wise analysis suggested that all the measurements distinguished the three conditions with p < 0.005. Moreover, as can be seen in Figure 8 the correlation coefficients of the intermittent coupling condition are narrowly distributed in the middle between those of the uncoupled and the coupled conditions (SC: CC = 0.62, IC: CC = 0.306, UC: CC = 0.006).

Figure S4.1. Comparing the different conditions of the synthetic data (stably coupled (SC), intermittently coupled (IC) and uncoupled (UC),) regarding strength of average CC.

In line with our main comparison between ‘coupled’ and ‘uncoupled’, CC and unembedded %REC resulted in the largest differences between the conditions. These findings support the use of the above measures in the evaluation of coupling even with less stable coupling over time.

Table S4.1. Welch’s one-way ANOVA comparing stably coupled, intermittently coupled and uncoupled conditions. U: unembedded, E: embedded.

| **One-Way ANOVA** | **F** | **df1** | **df2** | **p** |
| --- | --- | --- | --- | --- |
| CC BPM | 2145.6 | 2 | 184 | < .001 |
| %REC(U) BPM | 650.8 | 2 | 187 | < .001 |
| %LAM(U) BPM | 34.1 | 2 | 195 | < .001 |
| %REC(E) BPM | 158.3 | 2 | 192 | < .001 |
| %LAM(E) BPM | 71.1 | 2 | 195 | < .001 |
|  | | | | |
