# Supplementary material for: Evaluating heart rate coupling with Multi-dimensional Recurrence Quantification Analysis (MdRQA) and Cross-Correlation (CC): Analytical decisions and implications
Source: PLoS One. 2026 Sep 16;21(9):e0356801. doi: 10.1371/journal.pone.0356801 (PMC13580983; doi:10.1371/journal.pone.0356801)
Supplement: Supplementary file 5 [file pone.0356801.s005.docx]

S5. Windowed Cross Correlation (WCC)

WCC is typically performed to address the autocorrelation that characterizes physiological signals. Autocorrelation could yield spurious cross correlation between signals and is usually accounted for in one of three ways – false-pair surrogate analysis, autoregressive models or WCC. In false-pair surrogate analysis the autocorrelation of the time series remains the same for both the real pairs and the false pairs, hence, the autocorrelation could not be the main source of the difference in the coupling levels between the false and the real pairs. Instead, the two other approaches aim to reduce the autocorrelation for the analyzed signal. When using WCC, one splits the signals into segments or windows that are, naturally, shorter than the original signal and thus exhibit less long-term autocorrelation. Applying this method, the researcher determines the window size and that way affects the range of the autocorrelation that is being diminished – longer window permit extended autocorrelation.

Since WCC is widely used in physiological synchrony research, in the following analysis, we cover the comparison between CC and WCC utilizing the synthetic heart rate data. In order to focus on one window size, we tested the linearity assumptions (homoscedasticity, linearity, and normality of residuals) for various window sizes with 50% overlap between consecutive windows. Table 10 shows that a larger window is associated with more frequent violations of normality and homoscedasticity assumptions, while the opposite trend is found for the linearity assumption. For our analysis, we chose the window size that yields the lowest percentage of total violations – 40 data points.

Table S5.1. For each cross correlation window size, the table displays the percentage of the time series which significantly violate the assumptions linear correlation. The alpha-level was set to 0.05 – accordingly, by chance we expect 5% of the data sets to fail the tests.

| **CC Window size** | **Normality** | **Homoscedasticity** | **Linearity** | **Total** |
| --- | --- | --- | --- | --- |
| 5 | 5.9 | 7.6 | 84 | 86.2 |
| 10 | 11.3 | 8.1 | 77.9 | 81.3 |
| 20 | 18.1 | 10.3 | 67.7 | 75.7 |
| 40 | 27.7 | 13.4 | 52.4 | 70 |
| 80 | 41.9 | 18.7 | 39.1 | 71.1 |
| 160 | 62.4 | 28.4 | 30.7 | 81.5 |
| 320 | 83.2 | 42.2 | 22.7 | 93 |
| No Windows | 99.3 | 64.7 | 17.3 | 100 |
|  | | | | |

Subsequently, we tested the difference between the ‘coupled’ and the ‘uncoupled’ conditions applying both CC and WCC. While both methods distinguish between coupled and uncoupled time series, the effect size found using CC is larger than the one for WCC. Regarding the use of WCC instead of CC, we do not have a clear suggestion. Beyond the stronger effect found using CC, utilizing the whole time series can improve the interpretability of the results due to the more linear coupling found in the assumption testing. Nevertheless, if autocorrelation is not addressed in a different manner (e.g. false-pair surrogate analysis), segmenting the data and applying WCC might be beneficial.

Table S5.2. Welch’s t-test comparing ‘coupled’ and ‘uncoupled’ conditions.

| **Coupled-Uncoupled** | **Statistic** | **df** | **p** | **Cohen’s d** | **Mean (Coupled)** | **Mean (Uncoupled)** |
| --- | --- | --- | --- | --- | --- | --- |
| CC | 62 | 151 | < .001 | 8.77 | 0.62 | 0.006 |
| WCC | 53.1 | 180 | < .001 | 7.51 | 0.45 | 0 |
| *Note.* Hₐ μ _Coupled_ ≠ μ _Uncoupled_ | | | |  |  |  |
